# Danger appraisal and pathogen-avoidance mechanisms in stigma towards severe mental illness: the mediating role of affective responses

**DOI:** 10.1186/s12888-022-03951-x

**Published:** 2022-05-12

**Authors:** Ana Chamorro Coneo, Edith Aristizabal Diazgranados, Olga Hoyos de los Rios, Daniela Aguilar Santander

**Affiliations:** Psychology department, Universidad del Norte, Barranquilla-Atlántico, Colombia

**Keywords:** Stigma, Severe mental illness, Pathogen avoidance, Danger appraisal

## Abstract

**Background:**

Stereotypes of dangerousness are common predictors of stigmatising attitudes towards Severe Mental Illness (SMI). However less is known about pathogen avoidance mechanisms underlying stigma towards SMI, specially in samples of non-industrialised societies of Latin America and the Caribbean. The primary aim of this study was to examine pathogen-disgust sensitivity and danger appraisal mechanisms in responses of stigma towards SMI.

**Methods:**

Cross-sectional design with convenience sampling. Using an online survey, volunteers at the Universidad del Norte in Colombia (*N* = 271) provided their sociodemographic data and completed the Three-Domain Disgust Scale (TDDS). Participants were randomised to different descriptions of someone with SMI that varied in terms of aggressiveness (with and without danger) and causes of the SMI. Then, following the attribution questionnaire (AQ-27), respondents reported affective and discriminatory responses to the person in the description.

**Results:**

Increased disgust sensitivity to pathogen stimuli resulted in stronger reports of anger (β = *.14*; *p* = .03), and fear (*β* = 0.27; *p* < 0.001). The relationship between disgust sensitivity and discriminatory responses was indirectly mediated by fear towards SMI (Bootstrapped CI =—.04,—.009). Dangerousness attributions in the description of SMI predicted stronger feelings of anger (*β* = .23; *p* = 0.001) and fear (*β* = .40; *p* < .001), as well increased support for coercion-segregation of SMI (*β* = .34; *p* = 0.04), but less intentions to help (*β* = -.26; *p* = 0.003). The relationship between dangerousness and support for coercion was mediated by fear (Bootstrapped CI = .72, 1.37) and anger (Bootstrapped CI = .06, .44), whereas pity (Bootstrapped CI = .03, .38) and fear (Bootstrapped CI = -1.39, -.69) mediated responses of support for coercion-segregation of SMI. Attributions about causes and personal responsibility were not significantly linked to stigma towards SMI (*p* > 0.05).

**Conclusions:**

Findings suggested that pathogen avoidance and danger appraisal systems interplay in the generation of discriminatory behaviour towards SMI. Anti-stigma programs and policy makers would benefit from introducing strategies that challenge stereotypes of dangerousness and unpredictability by promoting positive contact with people with SMI.

## Background

Psychiatric and neurological disorders account for 30% of non-fatal disease burden worldwide [1]. Severe Mental Illnesses (SMI) are significant contributors to morbidity and disability, both due to the debilitating symptoms associated with these disorders and the stigmatisation that people with SMI must endure [2]. The disability associated with stigma obstructs social relationships, access to quality healthcare, housing possibilities and employment [3–5]. Stigma towards SMI has been reported cross-culturally [6], but studies testing explicative models and the effectiveness of anti-stigma strategies have mostly used samples from industrialised societies, leaving population from the global south heavily underrepresented [7].

From an evolutionary psychology perspective, behaviours are motivated by emotional systems that were shaped by natural selection to solve key adaptive problems, including those associated with socialisation. In the presence of stimuli that indicate danger, fear motivates cognitions and behavioural responses that may prompt the individual to seek protection (e.g., distancing from potentially dangerous peers) [8]. Conversely, affective responses of pity and compassion help facilitate group bonding and interpersonal repair [9]. Literature proposes that SMI triggers in the public a distinct pattern of affective responses, which are different to that occurring in response to other mental health conditions [10]. For instance, a study by Link et al. [11] showed that individuals with schizophrenia and alcohol dependence evoked fewer positive feelings and more fear and anger compared to individuals with depression.

Emotions like disgust have recently been explored in relation to different forms of stigma and prejudice [6]. Disgust is a core emotion that helps humans solve selection problems associated with avoidance of pathogen contamination in a literal sense (e.g., contact body fluids), but it is also evoked in response to social cues, such as peers with stigmatising attributes, including those with mental illnesses [12]. Evolutionary models suggest that disgust systems in stigma may serve a function in maximising survival of the individual by promoting (i) the avoidance of social interactions with peers that offer limited social gain; (ii) the maintenance of exploitative relationships towards certain minority groups and dominance over resources; and (iii) the reduction of the probability of being contaminated through contact with potential pathogens also known as behavioural immune system or BIS [13, 14]. Disgust seems to be a significant predictor of different forms of stigma and prejudice (e.g., ethnic minorities [15] and people with obesity [16]), but it is yet to be explored whether responses towards SMI obey a pathogen avoidance function.

Individual differences in the way people experience disgusting stimuli may influence stigmatising responses, particularly those resulting in avoidance and exclusion [17]. In Dawydiak et al. [12], higher levels of disgust sensitivity to pathogen cues (i.e., contact with highly contagious stimuli) predicted stronger stigma responses toward various mental health conditions (i.e., skin picking disorders, sexual sadism disorder and schizophrenia). However, direct comparisons showed non-significant results for the relationship between pathogen disgust sensitivity and stigma toward a person with schizophrenia. The limitations of correlation analysis in Dawydiak et al. [12], restrict the establishment of causal relationships, though future research efforts would benefit from investigating pathogen-avoidance mechanisms underlying stigma towards SMI.

In addition to the evolutionary psychology perspective, the social cognitive model has provided a compelling explanatory basis for understanding stigma as a by-product of information processing systems. Public stigma towards SMIs comprise three components: stereotypes, prejudice, and discrimination [18]. Stereotypes are cognitive schemes about the attributes, behaviours, and characteristics of a group member. Stereotypes about people with SMIs include dangerousness, unpredictability, and the belief that they are responsible for their symptoms (i.e., attribution of cause and personal responsibility). When people endorse these stereotypes, affective responses (e.g., fear, disgust) may lead to negative evaluations of the stigmatised target, known as prejudice. In turn, the behaviour resulting from this evaluation has been termed discrimination, and it is commonly measured as avoidance and social distance [19–21]. Other forms of discrimination include withholding help, hostility, public endorsement of mandatory custodial treatment and exclusion from the community (coercion and segregation) [22].

A comparative analysis of attitudes toward mental health conditions over five decades suggested a significant increase in the perception of dangerousness associated with people with SMI, especially if it is severe or serious [23]. Stereotypes of aggressiveness increase responses of fear, ultimately leading to greater desire for social distance and increased support for punitive treatment of people with SMIs, this mechanism is known as danger appraisal [22, 24]. The mediating role of affective responses (i.e., fear, anger, and pity) in the relationship between stereotypes and discrimination has received some support in the literature [25, 26], which has important implications to the development of anti-stigma strategies that effectively reduce discrimination. For instance, by promoting representations of SMI that do not conform to stereotypes of violence and dangerousness.

According to the attribution model [19], discriminatory behaviour towards SMIs follow a cognitive-emotional process: upon encountering a person with a SMI, people rely on information about dangerousness, but also about causal attributions (whether the person causes or has control over the SMI) to determine the level of responsibility of the target. When the SMI is attributed to causes that are "controllable" by the individual with the disorder (e.g., drug abuse), the individual is more likely to be perceived as responsible for his or her medical condition, leading to increased discriminatory responses. However, beliefs about what causes SMI are also influenced by the observer’s cultural and religious background. For instance, causal beliefs that are common to various Christian denominations include demonic possession, or a divine punishment. Consistently, religious treatments for SMI are oriented to address a spiritual weakness, through faith and prayer, rather than through traditional evidence-based alternatives [27]. Thus, it is possible that attitudes toward people with SMI are influenced by these beliefs about the aetiology of SMI and result in increased stigma [28]. Performing cross-cultural validations of the attribution model is key to developing anti-stigma strategies aimed at targeting specific attributions that result in responses of discrimination.

Affective (e.g., fear, anger) and behavioural responses (e.g., helping or avoiding) towards individuals with SMIs also rely on beliefs about personal responsibility of the person with SMI. When the person with SMI is not believed to be responsible for their mental health condition the public shows greater acceptance of the person with a SMI, which may include feelings of pity and increased likelihood of offering help [19, 25]. However, a recent literature review testing the effectiveness of anti-stigma strategies showed that increasing the endorsement of beliefs about biogenetic causes of mental illness did not reduce stigma, but rather increased it in 10 out of 11 articles [29].

Based on the interplay of cognitive and affective factors that predict stigma towards SMI, a few strategies have been formulated to reduce beliefs and challenge stereotypes about people with SMI (for a meta-analysis see Corrigan et al. [30]). Literature indicates that compared to education and protest, contact-based strategies are more effective at tackling negative stereotypes and improving attitudes towards people with SMI [31]. Familiarity with SMIs may range from viewing representations of SMIs in media outlets, to sharing colleagues, friends, and family members with SMIs [19, 32]. When the contact is positive (e.g., there are shared interests) and the stigmatised person challenges predominant stereotypes about the health condition or attribute, attitudinal and emotional responses in the public improve. Furthermore, the effectiveness of contact is greater when it involves a direct interaction, as opposed to media presentation formats [30]. If beliefs about what causes the mental health condition are not relevant predictors of stigma, trying to change or intervene these beliefs would not suffice to change affective and behavioural responses of stigma towards SMI. It is vital for anti-stigma strategies and public policy to target the specific attributes that result in stigma reduction to achieve effective results.

### The present study

This is the first study to date using evolutionary psychology and social cognitive perspectives to investigate stigma towards SMI in a sample of Colombian participants. Firstly, following the pathogen avoidance hypothesis of stigma or BIS, it was anticipated that stronger sensitivity to disgust (pathogen specific domain) would predict more negative affective responses (i.e., more fear and anger, but less pity; H1a), but also greater endorsement of personal responsibility (H1b) and increased discriminatory intentions towards SMI (i.e., support for coercion and intentions to help; H1c). It was also investigated wether affective responses of fear, anger and pity towards SMI would mediate the relationship between disgust sensitivity and discriminatory responses (H1d). Congruently with literature supporting the anti-stigma effect of contact, hypothesis two predicted that increased familiarity with SMI would decrease responses of stigma towards SMI (H2). The third hypothesis, based upon the attribution theory [19], expected that dangerousness and causal attributions would increase perceptions of personal responsibility, fear, anger, and intentions of coercion-segregation towards people with SMI. In contrast, feelings of pity and intentions to help would likely decrease with attributions of danger and control over symptoms (H3). Based on Corrigan’s findings, it was hypothesised that the relationship between causal attributions and discriminatory responses would be mediated by personal responsibility beliefs, but the relationship between dangerousness and discriminatory responses would be partially or fully mediated by fear, anger, and pity (H4).

## Methods

### Design and procedure

This study used a cross-sectional between-group design to investigate the effect of dangerousness, causal attributions, and disgust sensitivity on affective responses (i.e., fear, pity, anger) and discriminatory intentions (to help/avoid and coerce or segregate) towards an individual with a SMI. A convenient sample of Colombian residents over 18 years of age (student and non-student), accessed the online questionnaire by clicking on a link distributed via social media posts (e.g., Instagram, Facebook) and university mail lists. Volunteers were able to access an online questionnaire built on the survey distribution platform SoSci, which was available to users via www.soscisurvey.de.

The first page of the online questionnaire presented information about the study and asked participants to confirm their consent to take part in the study. The next page asked respondents to provide socio demographic information such as age, sex, religious affiliation, and relationship status. Then, participants completed a series of Likert-type questionnaires measuring disgust sensitivity and familiarity with SMI. Immediately after, respondents were randomly allocated to read vignettes portraying the story of “Juan”, a 30-year-old person with a diagnosis of schizophrenia [19]. Each participant was presented with one of four possible descriptions of “Juan” that varied in levels of dangerousness (danger appraisal) and attributions of control over the mental health problem: (1) Fifty-four respondents viewed a description of Juan without attributions of danger (e.g. “Juan is never dangerous”); (2) sixty-nine participants were presented with a vignette about Juan showing aggressiveness (“…he attacked a person in the emergency room”); (3) further 72 people received a description of Juan as aggressive but without controllability of cause (“Juan’s SMI was the result of a traffic accident”); and 76 participants were presented with a story in which Juan is dangerous and somehow responsible of his SMI (“Juan’s SMI is the result of substance abuse”). Following the presentation of vignettes, participants responded questionnaires measuring personal responsibility beliefs (i.e., whether Juan was responsible for the SMI or not), affective (i.e., anger, fear, pity) and discriminatory responses towards SMI (i.e., intention to help and coercion-segregation). The final part of the questionnaire debriefed and thanked respondents for their participation.

A pilot testing with a separate sample of 21 participants was conducted before initiating data collection for the full study. Pilot analysis findings corroborated that the wording of all the items composing the questionnaire were comprehensible for participants, and that there were no evident errors in the distribution of scores. The questionnaire for the full study was launched in the months of March and May 2021, in which 1198 volunteers clicked on the questionnaire’s link, but only 271 participants completed the questionnaire until the end, there was no missing data out of this remaining sample. Although completing the questionnaires was completely voluntary, in exchange for taking part in the study, participants were offered the chance to be included in the raffle prize of two 100,000 COP (26 USD) supermarket vouchers. Contact details of those choosing to take part in the raffle were set to be stored in a separate file to the responses of other sections of the questionnaire to ensure full anonymity of the data set. This study was reviewed and approved by a local university’s ethics committee (Ref. 225), and it complies with research standards for experiments with human participants.

### Sample size calculation

Prior the study, a power analysis was conducted using the software G*Power 3.1. Using as reference the effect size reported in Corrigan et al. [19](R^2^ = 0.73), the sample size required for a linear multiple regression with 15 predictors, an alpha of 0.05 and a power of 0.95, was calculated [33]. Results showed that a minimum of 199 participants would be needed to detect a medium effect size 95% of the time.

### Measures

#### Demographic data

Participants provided basic information about their age, sex, relationship status, religious affiliation, and social stratification status.

#### Familiarity with mental illness

The Level of Contact Report [32] was used to assess participant’s previous experience with SMI. This instrument is composed of seven items with “Yes (1)/ No (0)” response options. Statements reference different levels of contact, including being colleagues, friends, or close relatives with someone with SMI. In this study, the internal reliability of this instrument was adequate (α = 0.68).

#### Disgust sensitivity

The three-Domain Disgust Scale (TDDS; [34] measures sensitivity to experience disgust in reference to moral, sexual and pathogen cues. Participants completed the Spanish version of the TDDS, validated by [35]. The scale contains 21 statements that participants rate in a 7-point Likert scale, ranging from not disgusting at all (0), to extremely disgusting (6). The internal reliability of the TDDS in this study was good to excellent for the overall scale (α = 0.80) and the constructs of sexual (α = 0.80), moral (α = 0.86) pathogen disgust sensitivity (α = 0.77).

### Dependent variable

#### Stigma towards SMI

Respondents completed a Colombian-Spanish adaptation of the Attribution Questionnaire (AQ-27; [19]. The 27-item questionnaire, which includes the seven familiarity items described above [32], was translated using the quality assurance technique Back-Translation. With the participation of two translators, the original AQ [19] was translated into Colombian Spanish by one translator, and then back into English by the second one. Both translated versions of the instrument were compared, and discrepancies in word choices were discussed between translators. Once differences in translations were reconciled, the resulting version of the Colombian-Spanish AQ was pilot tested (*N* = 21) with the other variables of this study. Pilot results showed good comprehension of the items amongst participants and adequate distribution of the scales’ scores. Other validations of the AQ-27 in Spanish speaking samples [36, 37] also yielded good and acceptable psychometric properties for this instrument.

The AQ-27 in Colombian Spanish comprised four vignettes describing the story of “Juan”, a man with a SMI. The story in each vignette was different regarding Juan’s aggressiveness and causes associated with the cause and exacerbation of his symptoms. Participants were randomly presented with one of these conditions, followed by 20 statements to be rated on a 9-point Likert scale (1 = not at all to 9 = very much). In this study, psychometric properties were good across the subconstructs of anger (α = 0.81), fear (α = 0.96), helping/avoidance intentions (α = 0.84) and support for coercion-segregation (α = 0.86). Subconstructs of personal responsibility beliefs (α = 0.60) and pity (α = 0.55) were acceptable.

### Statistical analysis

Analyses were conducted using SPSS version 26. First, data from 271 participants underwent descriptive analysis and means were compared across manipulated conditions. Then, a series of biserial correlation analyses were conducted to test the relationship between variables and check for multicollinearity in the dataset. To ensure that the assumptions for multivariate regression analysis were met, further analysis included tests of independence of residual (Durbin-Watson = 2.03), homoscedasticity and normality.

To test hypotheses 1–3, a series of ordinary least squares (OLS) regression equations were conducted. Following the attribution model of stigma toward mental illness [19] variables were entered in the regression analyses in different blocks. The first block was composed of individual differences in sociodemographic characteristics and levels of disgust sensitivity. After that, a second block with dangerousness and causal attributions was entered, followed by a third block with personal responsibility beliefs. The regression models for discriminatory responses towards SMI (i.e., intentions to help and support for coercion-segregation) included a fourth block with affective responses of pity and anger and fear (block 4). To address hypothesis 1d and 4, variables with *p*-values below 0.05 were further tested for mediation using the PROCESS macro extension on SPSS version 3.3. Mediation analyses (Model 4) used bootstrap sampling with 5,000 replications and 95% confidence interval (CI) to test for the indirect effect of personal responsibility beliefs and affective responses (e.g., fear, anger, pity) on discriminatory responses towards SMI. An indirect effect was confirmed if the results of the CI did not include zero.

## Results

The sample in this study (*N* = 271) had a mean age of 32 years (SD = 14.06; range 18–79) and was composed predominantly of females (*n* = 188). Eighty-six participants (*n* = 86) reported being students, but mostly the sample included people of the public that engaged in income generating activities (*n* = 128). Socioeconomic status of participants included all levels, however the highest stratifications (5—6) were the least represented group (*n* = 41). Participants were in its majority single (*n* = 174) and from the Northern region of Colombia (*n* = 238). The Christian faith was the most reported religious affiliation, 133 respondents identified themselves as Catholics, further 43 were Christian protestant, and 88 reported no religious affiliation. See Table 1 for sociodemographic details.

**Table 1:** Sociodemographic details (*N* = 271)

Group	Frequency	Percentage
Sex		
Female	188	69.4
Male	82	30.6
Social Stratification Status		
1–2 (Low)	105	38.7
3–4 (Medium)	122	45
5–6 (High)	41	15.1
Religion		
Catholic	133	49.1
Protestant	43	15.9
No religious affiliation	88	32.5
Other	7	2.6
Occupation		
Part-time job	11	4.1
Full-time job	74	27.3
Unemployed	43	15.9
Freelance or business owner	42	15.5
Student	86	31.7
Retired	5	1.8
Relationship status		
Single	174	64.2
Married	69	25.5
Divorced	7	2.6
Free union or Living w/ significant other	17	6.3

The correlation coefficients between the main variables included in this study can be found in Table 2. Tables 3 and 4 shows the results of the OLS regression equations used to test hypotheses 1–3. Findings showed that sociodemographic differences were significant predictors of certain aspects of stigma towards SMI. Female participants showed significantly more pity than male participants, *B* = 0.52, β = *0.14, t* (12, 265) = 2.14, *p* = 0.03, and younger respondents showed greater likelihood to help someone with SMI, B = -0.026, *β* = -0.17, *t* (15, 262) = -2.78, *p* = 0.008. Participants with a partner reported weaker pity feelings compared to their counterparts without a relationship, *B* = -0.64, *β* = -0.18*, t* (12, 265) = -2.38, *p* = 0.01. Interestingly, while there was no significant effect of age, sex or relationship status on any of the other measurements of discrimination, findings indicated that compared to non-religious respondents, people with religious affiliation reported stronger feelings of fear, *B* = 0.82, *β* = 0.15, *t* (12, 265) = 2.51, *p* = 0.01, less pity, *B* = -0.49, *β* = -0.13, *t* (12, 265) = -2.09, *p* = 0.04, and more support for the coercion-segregation of people with SMI, *B* = 0.47, *β* = 0.11, *t* (15, 262) = 2.18, *p* = 0.03.

**Table 2:** Bivariate correlation matrix and descriptive statistics (*N* = 271)

Variable	M	SD	1	2	3	4	5	6	7	8	9
1. AQ: Familiarity	2.27	1.81									
2. AQ: Personal responsibility beliefs	3.28	1.54	.009								
3. AQ: Pity	6.26	1.70	.012	-.054							
4. AQ: Fear	4.25	2.53	-.085	.042	.294**						
5. AQ: Anger	2.86	1.74	-.069	.170**	.148*	.573**					
6. AQ: Help/Avoid	6.13	2.10	.175**	.006	.033	-.488**	-.350**				
7. AQ: Support for Coercion/Segregation	3.68	2.05	-.076	.127*	.224**	.693**	.511**	-.583**			
8. TDDS: Pathogen	25.45	8.40	-.055	.011	.111	.219**	.144*	-.177**	.217**		
9. TDDS: Sexual	22.56	9.99	-.026	.037	.044	.029	.051	-.097	.071	.460**	
10. TDDS: Moral	28.91	10.03	.017	.052	.154*	-.052	.005	.004	-.042	.151*	.214**

^**^ *p* < 0.01, * *p* < 0.05

**Table 3:** Standardised OLS Regression Coefficients for affective responses

Variable	Personal responsibility		Pity				Anger				Fear			
	(1)		(2)		(3)		(4)		(5)		(6)		(7)	
	***B***	**SE**	***B***	**SE**	***B***	**SE**	***B***	**SE**	***B***	**SE**	***B***	**SE**	***B***	**SE**
1. Age	.03	.00	-.11	.01	-.11	.01	-.08	.01	-.08	.01	-.01	.01	-.01	.01
2. Sex (Female = 1)	-.03	.22	.14*	**.24**	.14*	**.24**	-.11	.26	-.11	.24	.08	.33	.08	.33
3. Religious affiliation (religious = 1)	.05	.21	-.13*	**.23**	-.13*	**.23**	.06	.25	.05	.24	.15*	**.32**	.15*	**.32**
4. Relationship status (with partner = 1)	.05	.24	-.18*	**.27**	-.17*	**.27**	-.01	.28	-.02	.27	-.06	.37	-.06	.37
5. TDDS: Pathogen	.03	.01	.10	.01	.11	.01	.14*	**.01**	.14*	**.01**	.27*	**.01**	.27**	**.01**
6. TDDS: Moral	.05	.00	.15*	**.01**	.15*	**.01**	-.01	.01	-.02	.01	-.10	.01	-.11	.01
7. TDDS: Sexual	.00	.01	-.05	.01	-.05	.01	-.00	.01	-.00	.01	-.16	.01	-.16	.01
8. Familiarity with SMI	.04	.05	.00	.05	.01	.05	-.01	.06	-.02	.05	-.01	.07	-.01	.07
9. Danger (No danger = 0)	-.07	.27	.11	.30	.11	.30	.23**	**.31**	.25**	**.31**	.39**	**.42**	.40**	**.42**
10. With control over SMI (No info = 0)	.37*	**.24**	.03	.27	.05	.29	.10	.28	.03	.30	.04	.38	.03	.40
11. No control over SMI (No info = 0)	-.05	.24	.05	.28	.05	.28	-.04	.29	-.03	.28	-.03	.38	-.03	.38
12. AQ: Personal responsibility beliefs					-.03	.07			.15*	**.07**			.03	.09
13. AQ: Pity														
14. AQ: Anger														
15. AQ: Fear														
*R*^*2*^	.16**		.11**		.11**		.12**		.14**		.24**		.25**	

^*^ *p* < .05 **, *p* < .001 **(two-tailed tests)

**Table 4:** Standardized OLS Regression Coefficients for discriminatory responses

Variable	Intention to help/avoid						Support for coercion-segregation					
	**(8)**		**(9)**		**(10)**		**(11)**		**(12)**		**(13)**	
	***B***	**SE**	***B***	**SE**	***B***	**SE**	***B***	**SE**	***B***	**SE**	***B***	**SE**
1. Age	-.17*	**.01**	-.17*	**.01**	-.17*	**.01**	-.06	.01	-.06	.01	-.03	.00
2. Sex (Female = 1)	.00	.29	.00	.29	.01	.27	-.02	.27	-.01	.27	-.06	.22
3. Religious affiliation (religious = 1)	-.09	.28	-.09	.28	-.00	.26	.18*	**.26**	.17*	**.26**	.11*	**.21**
4. Relationship status (with partner = 1)	-.04	.32	-.04	.32	-.04	.29	-.06	.30	-.07	.30	-.03	.24
5. TDDS: Pathogen	-.16*	**.01**	-.16*	**.01**	-.05	.01	.25**	**.01**	.25**	**.01**	.09	.01
6. TDDS: Moral	.09	.01	.09	.01	.01	.01	-.07	.01	-.08	.01	-.04	.01
7. TDDS: Sexual	.01	.01	.01	.01	-.04	.01	-.06	.01	-.06	.01	.01	.01
8. Familiarity with SMI	.12*	**.06**	.12*	**.07**	.11*	**.06**	-.00	.06	-.01	.06	.00	.05
9. Danger (No danger = 0)	-.26**	**.36**	-.26**	**.37**	-.09	.35	.34**	**.34**	.35**	**.34**	.12*	**.29**
10. With control over SMI (No info = 0)	-.03	.33	-.04	.35	-.03	.32	.10	.30	.06	.32	.03	.26
11. No control over SMI (No info = 0)	-.01	.33	-.01	.33	-.04	.30	-.00	.31	.00	.30	.01	.24
12. AQ: Personal responsibility beliefs			.00	.09	.04	.08			.09	.08	.06	.06
13. AQ: Pity					.18*	**.07**					.03	.05
14. AQ: Anger					-.09	.07					.15*	**.06**
15. AQ: Fear					-.43**	**.06**					.48**	**0.05**
*R*^*2*^	.17**		.17**		.34**		.24**		.25**		.52**	

^*^ *p* < .05 **, *p* < .001 **; *SE* Standard error

As stated in hypothesis 1a, participants with higher levels of disgust sensitivity to pathogen cues also showed greater feelings of anger, *B* = 0.03, *β* = 0.14, *t* (12, 262) = 2.14, *p* = 0.03, and fear towards SMI, *B* = 0.08, *β* = 0.27, *t* (12, 265) = 4.50, *p* < 0.001, but in the case of pity towards SMI, it was the moral disgust sensitivity domain that appeared to be a significant predictor, *B* = 0.02, *β* = 0.15, *t* (12, 265) = 2.46, *p* = 0.01. Contrary to anticipated outcomes in hypothesis 1b, disgust sensitivity did not predict stronger personal responsibility towards a person with SMI (*p* < 0.05). See Tables 3 and 4 for details at the end of the document text file.

**Fig. 1:**
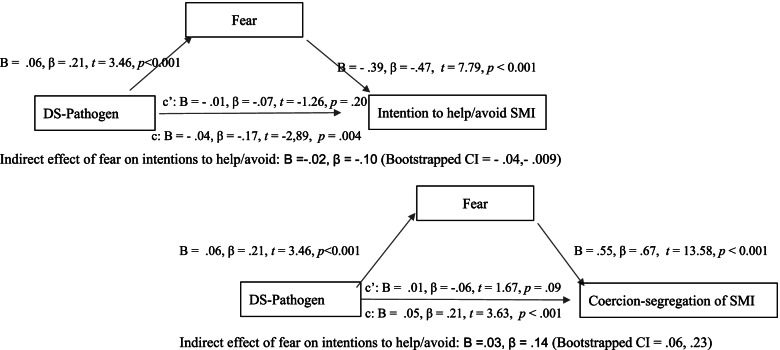
Testing fear as a mediator of the association between pathogen disgust sensitivity and discriminatory responses towards SMI

In regard to the behavioural measures (H1c), it was observed that stronger pathogen disgust sensitivity predicted more support for the coercion-segregation treatment of SMI, *B* = 0.084, *β* = 0.18, *t (*12, 262) = 3.26, *p* = 0.001, however, this effect ceased to be significant when fear was included in the regression model, *B* = 0.02, *β* = 0.09, *t (*15, 262) = 1.73, *p* = 0.08. Similarly, greater pathogen disgust sensitivity predicted more intentions to avoid (less intentions to help) someone with SMI, *B* = -0.04, *β* = *-*0.13, *t (*12, 262) = -2.20, *p* = 0.03, but this relationship became non-significant when fear was incorporated in the model, *B* = -0.01, *β* = *-*0.05, *t (*15, 262) = -0.94, *p* = 0.34. These results suggested an indirect mediation of fear responses in the relationship between pathogen disgust sensitivity and discriminatory outcomes, which was indeed corroborated by further mediation analyses (H1d). Greater pathogen specific disgust sensitivity was associated with greater intentions to avoid SMI (Bootstrapped CI: -0.07, -0.01), but this relationship was fully mediated by responses of fear towards SMI (Bootstrapped CI =—0.04,—0.009), and the model explained 24% of the variance in helping (avoidance) intentions towards an individual with SMI, *F* (2, 268) = 35.35, *p* < 0.001, R^2^ = 0.24. Analogously, fear fully mediated the relationship between pathogen specific disgust sensitivity and endorsement of coercion-segregation of SMI (Bootstrapped CI = 0.06, 0.23), and this model accounted for 48% of the variance in support for coercion-segregation, *F* (2, 268) = 119.01, *p* < 0.001, R^2^ = 0.48. See Fig. 1.

Hypothesis two (H2) was only partially supported, familiarity with SMI did not result in improved affective responses (*p* > 0.05), nor did it reduce support for coercion segregation (*p* > 0.05), however, it did predict greater intentions to help someone with SMI, *B* = 0.13, *β* = 0.11, t *(*15, 262) = 2.07, *p* = 0.04.

In agreement with hypothesis three (H3), participants who read the vignette portraying Juan’s illness as the result of drug overuse (controllability) were more likely to think that he was responsible for the cause and onset of his SMI, B = 1.29, *β* = *37, t* (11, 265) = 5.25, *p* < 0.001. However, attributions about cause and control of the SMI were not significant predictors of affective (i.e., pity, anger or fear) nor discriminatory responses toward SMI.

The equations two to thirteen show the effect of danger attributions on affective and discriminatory responses toward SMI (i.e., intentions to help and support for coercion-segregation). No significant effect of dangerousness was found on reports of pity (*p* > 0.05). However, as anticipated in hypothesis three (H3), it was observed that danger attributions were associated with significantly stronger feelings of anger towards SMI, B = 1.11, *β* = *25, t* (12, 262) = 3.51, *p* = 0.001, and fear, B = 2.53, *β* = *0.40, t* (12, 265) = 6.03, *p* < 0.001. Also, increased support for coercion-segregation of SMI were reported when Juan’s story included dangerousness B = 0.62, β = *0.12, t (*15, 262) = 2.13, *p* = 0.04. Congruently, participants reported significantly less intentions to help when dangerousness depictions were included in the vignette, B = -1.07, *β* = *-0.26, t (*12, 262) = -3.00, *p* = 0.003, but when including fear in the model, the relationship between dangerousness and intentions to help became non-significant, B = -0.03, *β* = *-0.09, t (*15, 262) = -1.46, *p* = 0.14, suggesting a potential mediation effect of fear in the relationship between fear and helping intentions towards individuals with SMI.

**Fig. 2:**
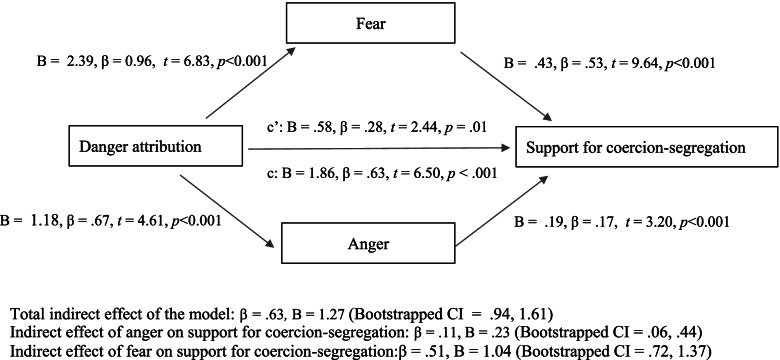
Testing fear and anger as a mediators of the association between danger attributions and support for the coercion-segregation of SMI

**Fig. 3:**
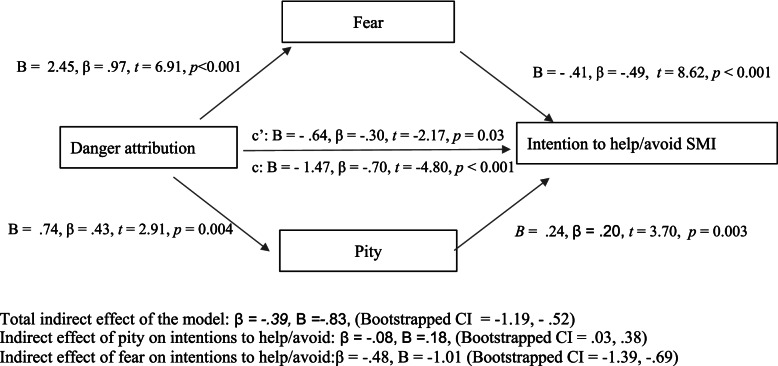
Testing fear and pity as a mediators of the association between danger attributions and intentions to help or avoid people with SMI

Lastly, further mediational analyses were conducted to address hypothesis four (H4). Results showed that affective responses of fear (Bootstrapped CI: 0.72, 1.38) and anger (Bootstrapped CI: 0.06,0.45) indirectly mediated the relationship between dangerousness attributions and support for coercive treatment of SMI (Bootstrapped CI: 0.44, 1.62), and this model explained 49% of the variance in support for coercive treatment of SMI, *F* (3, 264) = 86, *p* < 0.001, R^2^ = 0.49. Pity was not included in the mediation analysis because dangerousness did not predict feelings of pity (B = 0.05, *t* = 1.63, *p* = 0.10), thus breaching an assumption for mediation analysis. See figs. 2 and 3 for details.

Affective responses of fear (Bootstrapped CI: -1.37, -0.68) and pity (Bootstrapped CI: 0.032, 0.38) indirectly mediated the relationship between dangerousness and intentions to help/avoid people with SMI (Bootstrapped CI: -1.18, -0.50), with 28% of the variance in helping intentions explained with this model, *F(*3, 267) = 35.49, *p* < 0.001, R^2^ = 0.28. Although greater feelings of anger predicted significantly fewer helping intentions in equation 11, with the introduction of the variable fear this relationship became non-significant, therefore making it unfit for its inclusion in the mediation analysis.

## Discussion

This study had a two-fold aim, first, to examine the role of disgust sensitivity systems in relation to known danger appraisal responses of stigma towards SMI (H1a-H1d); and second, to test attribution models of stigma [19] in a sample of Colombian participants (H2-H4). Findings indicated that disgust sensitivity systems were associated with stronger affective and behavioural responses towards SMI, but emotions of fear fully mediated the effect of disgust sensitivity over discriminatory intentions. In addition, observers responded more negatively towards people with SMI (i.e., stronger feelings of anger and fear, and more discriminatory responses) when the description of SMI included information of dangerousness, but attributions of control and personal responsibility had little effect over stigmatising responses in this sample. Further mediation analysis showed that the relationship between dangerousness and discriminatory responses was indirectly mediated by feelings of fear, anger, and pity.

As hypothesised, the variable of disgust sensitivity appeared to play a significant role in the interplay of factors associated with stigma towards SMI. Disgust sensitivity specific to pathogen cues predicted stronger responses of anger and fear towards SMI [12]. Furthermore, the relationship between pathogen disgust sensitivity and discriminatory responses was fully mediated by fear towards SMI. Thus, suggesting that pathogen-avoidance systems in stigma towards SMI may operate within fear responses to danger cues (e.g., SMI danger attributions), which ultimately would increase avoidance and discrimination [11]. Literature would benefit from further examining the function of stigma as an evolved mechanism of protection against potential contamination and danger, for instance, by testing the effect of induced disgust in stigmatising responses toward SMI.

Another interesting finding was that rather than pathogen-specific, it was the moral-specific domain of disgust sensitivity that predicted stronger responses of pity. It may be that the disgust sensitivity system that responds to stimuli resembling moral transgressions also influences responses related to group bonding and interpersonal repair [9, 10]. These findings corroborate that pathogen disgust sensitivity systems operate within mechanisms that maximise survival of the individual through avoidance (BIS; [12–14]), but other domains of disgust (i.e., moral) could also influence behaviours that are antagonistic of stigma, such as prosocial behaviour [38]. Further investigating the role of specific disgust domains within stigmatisation responses would benefit the current understanding about the evolutionary roots of human socialisation.

As anticipated, participants that were more familiar with SMI reported more likelihood to help others with SMI, though unlike previous reports with American samples [19], greater contact with SMI had no effect over other measures of stigma. This finding was unexpected, given the extensive body of literature supporting the anti-stigma effect linked to contact [30–32]. A possible interpretation could be related to the quality of participants' relationship with SMI, as close contact does not necessarily indicate positive contact. For instance, reports from family members of people with SMI in Colombia included feelings of grief and denial upon receiving a relative’s diagnosis [39]. Further exploration of the quality of the contact with SMI may help further clarify the relationship between familiarity, causal attributions, and stigma toward SMI.

The third hypothesis of this study predicted that beliefs about external causes of the SMI (e.g., caused by an accident or biogenetic factors) would be associated with reduced stigma compared to those attaching responsibility to the person with the SMI (e.g., drug abuse related). However, beliefs about whether a person with SMI had control over the cause or onset of his mental health condition or not had little effect on the way people responded to SMI. While these findings were not anticipated, findings go in line with previous reports showing that increased knowledge about biogenetic causes of SMI (i.e., external cause) did not decrease stigma toward mental illness, but rather increased it [29]. These findings indicate that stigma towards SMI is mediated by affective responses rather than personal responsibility beliefs, thus suggesting a danger appraisal mechanism in observers’ responses toward SMI.

A factor that may possibly be influencing these contrasting results may be associated with differences in sociocultural backgrounds between participants of this study and the American sample in Corrigan et al., 2003 [19]. Mascayano et al., 2016 [4] suggest that Hispanic and Latin American societies have specific cultural orientations toward family and community that may serve as a buffering factor for stigma. In addition, most participants in this study reported following Christian creeds that are known for endorsing beliefs about spiritual aetiologies of SMIs [27, 28]. Compared to non-religious people, participants with a reported religious affiliation showed greater stigma, which may have influenced non-significant results regarding the relationship between causal attributions and affective and discriminatory responses toward SMI. The literature would be strengthened with supplemental analyses about how different dimensions of religiosity and other culturally specific characteristics influence stigma, so that they can be incorporated into anti-stigma efforts that are culture specific.

The interpretation of these findings must consider some limitations. First, the convenience sampling technique used in the study risks generalisation of results to the general Colombian public. For instance, there was a disproportionate ratio of females (69%), and participants from the Caribbean region made up over 80% of the sample, which may incur underrepresentation of certain subgroups of the population. Stricter sampling techniques such as stratified randomised sampling would aid representativeness in upcoming studies.

Second, because stigma and prejudice responses challenge social values of egalitarianism, people often respond in ways that adjust to expectations about the subject rather than how they “actually feel” [40]. Social desirability bias could be a limitation, because all the measures used in this study had a self-report format and there was no control over potential social desirability effects by using a social desirability scale. Future literature would benefit from introducing alternative measures of stigma with less face value, such as physiological responses, behavioural measures (e.g., proximity) or masked self-report questionnaires (e.g., error choice test; [41]. Finally, the AQ-27 restricted responses to a specific individual (i.e., “Juan”), and only accounted for one mental health condition (i.e., schizophrenia). Examining observers’ responses to different targets (e.g., a female with depression or anxiety) would help to better understand the phenomenon of stigma toward different levels of SMI.

Despite these limitations, this study’s findings provide valuable insights that contribute to the understanding of stigma towards SMI under evolutionary and social psychology perspectives. Findings indicated that BIS mechanisms influence behavioural responses of stigma towards SMI, via increased responses of fear (danger appraisal). It may be that pathogen disgust sensitivity systems interplay with danger appraisal mechanisms in the generation of responses towards SMI. Stigma towards SMI is likely part of a mechanism evolved to protect humans from potential contamination and maximise chances of survival, but disgust systems may also influence other aspects of social exchange, for instance, motivating behaviour associated with interpersonal repair or pity. Under the light of these findings, practitioners and policy makers would benefit from introducing anti-stigma strategies that reduce emotions of fear and foster prosocial emotions of empathy. For instance, by introducing affirmative action policies that increase non-violent representations of people with SMI in the media and more inclusion of people with SMI within the community.

## Data Availability

Data is not available for online access, however readers who wish to gain access to the data can write to Junior lecturer Dr Ana Maria Chamorro Coneo at chamorroa@uninorte.edu.co with their requests. Access can be granted subject to the Institutional Review Board (IRB) and following research collaborative agreement guidelines. This is a requirement mandated by our IRB body.

## Abbreviations

AQ: Attribution Questionnaire
SMI: Severe Mental Illness
TDDS: The three-Domain Disgust Scale
